# A unified relationship for evaporation kinetics at low Mach numbers

**DOI:** 10.1038/s41467-019-10209-w

**Published:** 2019-05-30

**Authors:** Zhengmao Lu, Ikuya Kinefuchi, Kyle L. Wilke, Geoffrey Vaartstra, Evelyn N. Wang

**Affiliations:** 1 Department of Mechanical Engineering, Massachusetts Institute of Technology, Cambridge, MA 02139 USA; 2 Department of Mechanical Engineering, University of Tokyo, Bunkyo, Tokyo, 113-8656 Japan

**Keywords:** Applied mathematics, Fluid dynamics

## Abstract

We experimentally realized and elucidated kinetically limited evaporation where the molecular gas dynamics close to the liquid–vapour interface dominates the overall transport. This process fundamentally dictates the performance of various evaporative systems and has received significant theoretical interest. However, experimental studies have been limited due to the difficulty of isolating the interfacial thermal resistance. Here, we overcome this challenge using an ultrathin nanoporous membrane in a pure vapour ambient. We demonstrate a fundamental relationship between the evaporation flux and driving potential in a dimensionless form, which unifies kinetically limited evaporation under different working conditions. We model the nonequilibrium gas kinetics and show good agreement between experiments and theory. Our work provides a general figure of merit for evaporative heat transfer as well as design guidelines for achieving efficient evaporation in applications such as water purification, steam generation, and thermal management.

## Abstract (web summary)

Evaporation plays a key role in applications such as cooling and desalination. Here, the authors experimentally demonstrated a unifying relationship between dimensionless flux and driving potential for evaporation kinetics under different working conditions.

## Introduction

**Fig. 1:**
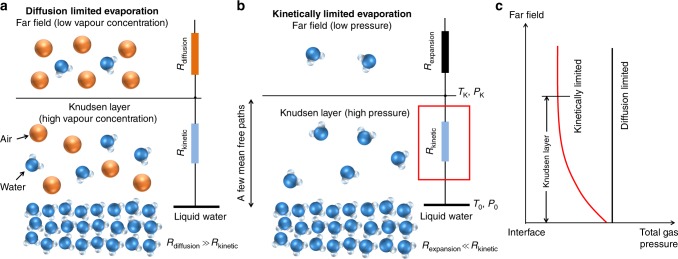
Physical picture of evaporation driven by different mechanisms. **a** Schematic of evaporation in air (not to scale): the far field vapour concentration is lower than the concentration at the interface and the transport resistance associated with gas diffusion (*R*_diffusion_) is much greater than the resistance across the Knudsen layer (*R*_kinetic_). **b** Schematic of evaporation in vapour (not to scale): the far field pressure is lower than the pressure at the interface and the transport resistance associated with gas expansion (*R*_expansion_) is much smaller than *R*_kinetic_, which allows us to focus on *R*_kinetic_ in this study (red box). **c** Sketch of total gas pressure distribution for diffusion limited and kinetically limited evaporation (not to scale): there is no variation in the total gas pressure when evaporation is diffusion limited; in kinetically limited evaporation, the evaporation is driven by the pressure difference which is mainly distributed across the Knudsen layer

Evaporation, a commonly found phenomenon in nature, is extensively used in water desalination^1,2^, steam generation^3^, and electronics cooling^4–6^. It also plays an important role in many fundamentally interesting physical systems such as diminishing sessile drops^7^ and growing vapour bubbles^8^. For evaporation systems operating in air, as in the case of multi-effect distillation^1^ and solar steam generation^3^, the interfacial flux is driven by the vapour concentration gradient between the interface and the far field (Fig. 1a). Previous studies demonstrated that the diffusion process limits the overall transport in this case^9,10^.

In many other applications, e.g., steam turbines^11^, vapour chamber thermal management devices^6^, and mechanical vapour compression desalination systems^2^, noncondensable gases are removed to create a pure vapour ambient, which eliminates the diffusion resistance and enhances the performance. The fundamental interfacial transport limit in these systems is dictated by the kinetics across a nonequilibrium gas region which inevitably forms near the evaporating surface^12^. This region, which is a few mean free paths thick, is known as the Knudsen layer and originates from the net flux across the interface disturbing the local thermodynamic equilibrium. For evaporation in a pure vapour ambient (Fig. 1b), there exists a pressure difference from the interface to the far field unlike diffusion limited evaporation (Fig. 1c), which facilitates the vapour flow. The gas expansion resistance beyond the Knudsen layer is usually insignificant for subsonic flows^13,14^ (see Supplementary Note 1). Consequently, it is possible to obtain fundamental insights into kinetically limited evaporation with vapour ambient evaporation.

While significant theoretical efforts have offered some insights into the gas kinetics in the Knudsen layer, experimental studies have proved difficult due to limitations in vapour removal^10,15^, interface temperature sensing^16,17^, thermal conduction^18,19^, and liquid supply^20,21^. Theoretically, the gas kinetic resistance (*R*_kinetic_) has been modeled by the Boltzmann transport equation (BTE) which governs the evolution of distribution functions of vapour molecules in the Knudsen layer^12,22^. Moment solutions^14,23^, semi-analytical results^12,24^ as well as numerical solutions^25–27^ of the BTE have been reported for the Knudsen layer problem. On the other hand, experiments have been limited due to various reasons. First, sensing the interface temperature can be difficult. Merely placing the sensor remotely does not give enough accuracy^17^ and simply inserting a thermocouple into the interface can disturb local evaporation^16^. Further, evaporation is often hindered by thermal conduction^18,19^ and viscous loss^20,21^ in the liquid, which makes the kinetically limited regime inaccessible.

In this work, we develop an ultrathin nanoporous membrane device to overcome previous challenges. With this experimental platform and assisted by nonequilibrium gas kinetics modeling, we elucidate a fundamental relationship that unifies evaporation kinetics under different working conditions.

## Results

### Experimental setup

**Fig. 2:**
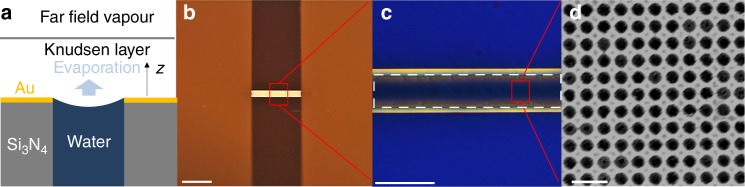
Nanoporous evaporation device. **a** Schematic of evaporation from a nanopore (not to scale). The gold layer is resistively heated to induce evaporation from a pinned meniscus in each nanopore. **b** Image of device with two gold contact pads connected by a free-standing membrane (<200 nm thick). The white scale bar is 2 mm. **c** Magnified view of free-standing membrane where the central part (white dashed box) is porous and coated with gold and the rest is impermeable and nonmetallic. The white scale bar is 500 μm. **d** Scanning electron microscope image of the nanoporous membrane with ≈140 nm diameter pores. The white scale bar is 400 nm

Figure 2a shows the nanodevice design that allows the liquid to wick into the nanopores with capillarity and the top corner of the pore creates a local energy barrier to prevent the liquid front from wetting the top membrane surface^28^. The free-standing membrane, connecting two electrical contact pads (Fig. 2b) is of thickness <200 nm, made of silicon nitride. The active part of the membrane, located in the middle (white dashed box in Fig. 2c), is coated with ≈40 nm gold. We defined a nanoporous pattern with pore diameter ≈140 nm and porosity ≈0.40 (>20,000,000 pores) in this active area using interference lithography^29^ (Fig. 2d), while the rest of the membrane is kept non-metallic and impermeable (see Supplementary Fig. 1 and Supplementary Note 2 for the fabrication process and Supplementary Fig. 2 for the cross-section image of the device). Evaporation is induced from the pinned interfaces in the nanopores through electrically heating the gold layer which also serves as a local resistive temperature detector (RTD) to address the typical temperature sensing issue. The combination of small pore diameter and membrane thickness minimizes resistances in thermal conduction and liquid supply, allowing us to probe kinetically limited evaporation (see Supplementary Note 3 for detailed design rationale).

**Fig. 3:**
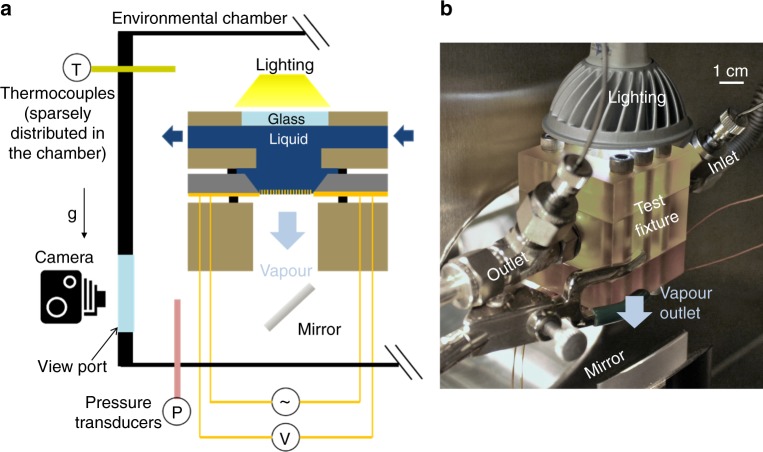
Experimental setup. **a** Schematic of device placed in a custom test fixture in an environmental chamber which allows for liquid feedthrough, electrical connection, and visualization (not to scale). **b** Image of the experimental setup

The reliability of this test platform was demonstrated in our previous work where evaporation was still limited by diffusion^10^. To induce kinetically limited evaporation, in this work, the device was placed in an environmental chamber with noncondensable gases removed to enable controlled vapour temperature and pressure of the far field (Fig. 3a, b). A custom test fixture interfaced the device to the liquid ports and electrical connections while facilitating visualization of the membrane surface during operation. Deionized water was used as the working fluid. To quantify the heat loss of the system, we fabricated a control sample with the same structure as the designed device without open pores, to make the active part impermeable. We determined the heat loss conductance of the system to be *C* = 4.7 ± 0.1 mW/K (see Supplementary Figs. 3 and 4 and Supplementary Note 4). During operation, the interfacial heat flux $\dot q{\prime\prime}$ was then recorded as

1 $$\dot q{\prime\prime} = \frac{{Q - C\Delta T}}{A}$$

where *Q* is the total Joule heating power, *A* is the pore area, and Δ*T* is the temperature rise of the membrane. *C*Δ*T* was generally less than 5% of *Q* in the present study. Equation (1) assumes that the interfaces were flat during the experiment, which is a result of the low hydraulic pressure loss across the ultrathin membrane (Supplementary Note 3). We regulated the ambient vapour pressures *P*_v_ by a temperature-controlled boiling canister degassed prior to the experiments. The vapour ambient temperature *T*_v_, on the other hand, was controlled by the heating power put into the chamber wall (see Supplementary Fig. 5 and Supplementary Note 5 for detailed experimental procedures). Throughout the experiment, the uncertainty in vapour pressure measurement was ±138 Pa, and the error of the interface temperature measurement was ±0.52 K (Supplementary Fig. 6 and Supplementary Note 6).

### Interfacial heat flux as a function of temperature

**Fig. 4:**
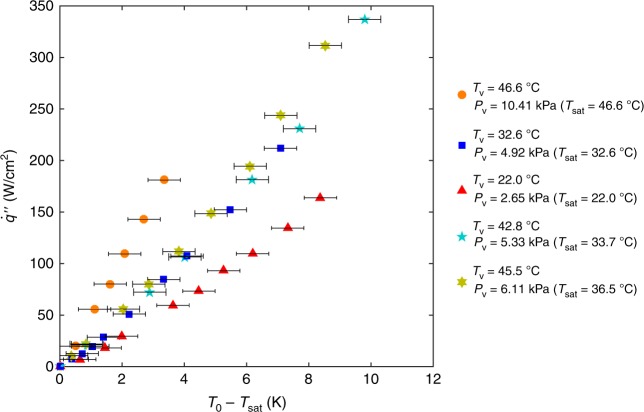
Experimental results of *q̇″* as a function of *T*_0_ − *T*_sat_ for select working conditions. The red triangles, dark blue squares, and orange circles represent the experimental data in saturated vapour for *P*_v_ = 2.65 kPa with *T*_v_ = *T*_sat_ = 22.0 °C, *P*_v_ = 4.92 kPa with *T*_v_ = *T*_sat_ = 32.6 °C, and *P*_v_ = 10.41 kPa with *T*_v_ = *T*_sat_ = 46.6 °C, respectively. The light blue pentagrams are for *T*_v_ = 42.8 °C, *P*_v_ = 5.33 kPa with *T*_sat_ = 33.7 °C and the yellow hexagrams are for *T*_v_ = 45.5 °C, *P*_v_ = 6.11 kPa with *T*_sat_ = 36.5 °C. Note that the light blue pentagrams are also for a sample with a different porosity (≈0.23) and pore diameter (≈106 nm). The error bars in the heat flux measurement are smaller than the symbol size and generally <2%. The temperature measurement error is 0.52 K in the present (see Supplementary Note 6 for detailed analysis)

Figure 4 shows *q̇″* as a function of *T*_0_ − *T*_sat_ for various working conditions, where *T*_0_ is the membrane temperature, and *T*_sat_ is the vapour saturation temperature associated with *P*_v_. In this study, the membrane temperature also represents the temperature of the evaporating surface (see Supplementary Note 3 for justification). Below, we use the subscript 0 to denote saturated vapour properties at *T*_0_. We implemented the correlation between pressure and temperature of saturated water vapour using the International Association for the Properties of Water and Steam Industrial Formulation 1997^30^. The red triangles, dark blue squares, and orange circles in Fig. 4 represent the experimental data in saturated vapour for *P*_v_ = 2.65 kPa with *T*_v_ = *T*_sat_ = 22.0 °C, *P*_v_ = 4.92 kPa with *T*_v_ = *T*_sat_ = 32.6 °C, and *P*_v_ = 10.41 kPa with *T*_v_ = *T*_sat_ = 46.6 °C, respectively. We also performed experiments where *T*_v_ > *T*_sat_ and the ambient vapour was unsaturated for: (1) *T*_v_ = 42.8 °C, *P*_v_ = 5.33 kPa with *T*_sat_ = 33.7 °C (light blue pentagrams) and (2) *T*_v_ = 45.5 °C, *P*_v_ = 6.11 kPa with *T*_sat_ = 36.5 °C (yellow hexagrams). In particular, in case (1), the ambient vapour pressure was similar to the 32.6 °C saturated case, but the vapour temperature was quite different. In case (2), the vapour temperature was close to the 46.6 °C saturated case while the vapour pressure was significantly different. In Fig. 4, data for both (1) and (2) are located close to the result for the 32.6 °C saturated case, indicating that kinetically limited evaporation is more sensitive to *T*_sat_ (or *P*_v_) rather than *T*_v_. This is generally because the speed at which the vapour moves from the interface to the ambient is more affected by *P*_v_ rather than *T*_v_. Moreover, given a superheat (*T*_0_ *− T*_sat_), an increasing *T*_sat_ results in higher heat fluxes or higher heat transfer coefficients (defined as *q̇″*/(*T*_0_ *− T*_sat_)) in Fig. 4.

### Modeling nonequilibrium gas kinetics

To obtain a more fundamental understanding from the experimental data, we modeled the nonequilibrium gas kinetics in the Knudsen layer. We first considered the boundary condition of BTE at the liquid–vapour interface, which is set by the evaporation coefficient and condensation coefficient (*σ*_e_ and *σ*_c_)^25,31,32^. The evaporation coefficient is defined as the ratio of molecules emitted from the interface to what the Maxwell-Boltzmann distribution predicts. The condensation coefficient is defined as the probability of incident molecules condensing into the liquid. At equilibrium conditions, *σ*_e_ = *σ*_c_ = *σ*^33^. As we focused on low Mach number evaporation, we carried on this equality. We solved the BTE in the Knudsen layer using the Direct Simulation Monte Carlo (DSMC) method^34^, with the variable soft sphere collision model^35^ and the Borgnakke-Larsen method^36^ to account for the temperature dependent properties and internal degrees of freedom of water molecules (see “Methods” for details of DSMC modeling). Previous theoretical studies mostly assumed the molecules to be monatomic, but more recently, Frezzotti^25,37^ showed significant differences of evaporation kinetics between polyatomic molecules and monatomic ones. This signifies the effect of the internal degrees of freedom of vapour molecules, which has generally not been properly considered in prior works when interpreting experimental data.

In this study, we nondimensionalized the interfacial heat flux *q̇″* as

2 $$\bar{\dot{q}}{\prime\prime} = \frac{{\dot q{\prime\prime}}}{{\rho _0u_{\mathrm{s}}\Delta h_{{\mathrm{lv}}}}}$$

Here, *ρ*_0_ is the density of saturated vapour at *T*_0_, *u*_s_ = (*γRT*_0_)^1/2^ is the vapour sonic speed evaluated at *T*_0_ where *γ* is the adiabatic index, and Δ*h*_lv_ is the enthalpy difference between two phases. In this study, we expect the pore size to have little effect on Δ*h*_lv_ at the 100 nm scale, as also found in our prior work^20^. It has been demonstrated in previous studies^14,23–25^ that the evaporation problem in the Knudsen layer can be characterized by the following three parameters: *P*_K_/*P*_0_, *T*_K_/*T*_0_, and *M*_K_. Here, *P*_K_, *T*_K_, and *M*_K_ are the pressure, temperature, and Mach number of the vapour adjacent to the Knudsen layer, respectively, and *P*_0_ is the saturation pressure at *T*_0_. Recognizing *q̇″* *=* *ρ*_K_*u*_K_Δ*h*_lv_ where *ρ*_K_ and *u*_K_ are the density and bulk velocity of the vapour adjacent to the Knudsen layer, we can rewrite Eq. (2) as

3 $$\bar{\dot{q}}{\prime\prime} = \frac{{\rho _{\mathrm{K}}u_{\mathrm{K}}}}{{\rho _0u_{\mathrm{s}}}}.$$

Assuming ideal gas behaviour, we have

4 $$\frac{{\rho _{\mathrm{K}}}}{{\rho _0}} = \frac{{P_{\mathrm{K}}}}{{P_0}}/\frac{{T_{\mathrm{K}}}}{{T_0}}.$$

and since *M*_K_ = *u*_K_/(*γRT*_K_)^1/2^,

5 $$\frac{{u_{\mathrm{K}}}}{{u_s}} = M_{\mathrm{K}}\sqrt {\frac{{T_{\mathrm{K}}}}{{T_0}}} .$$

Then clearly, $\bar{\dot{q}}{\prime\prime}$ is a function of *P*_K_/*P*_0_, *T*_K_/*T*_0_, and *M*_K_. In our simulation, for a given *σ*, one of these three parameters uniquely determines the other two, which was also reported in previous literature^14,24,25^. Equivalently, each of the three parameters is a function of Δ*P*/*P*_0_ where Δ*P* = *P*_0_ − *P*_K_. Consequently, we can write

6 $$\bar{\dot{q}}{\prime\prime} = f_\sigma \left( {\frac{{\Delta P}}{{P_0}}} \right).$$

In this form, Δ*P*/*P*_0_ can be considered as the dimensionless driving potential with *q̇̅″* being the dimensionless flux. Based on Eqs. (2) and (6), it also becomes obvious why the heat transfer coefficient is enhanced for higher *T*_sat_ as *ρ*_0_ increases sharply with temperature.

### Unified relationship for evaporation kinetics

**Fig. 5:**
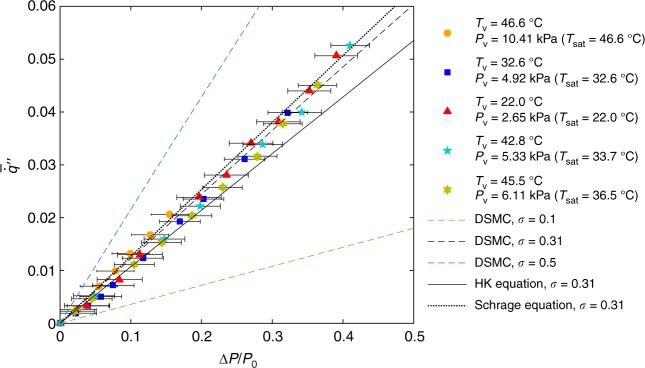
Dimensionless interfacial flux *q̇̅*″ as a function of the dimensionless driving potential Δ*P/P*_0_. The red triangles, dark blue squares, and orange circles represent the experimental data in saturated vapour for *P*_v_ = 2.65 kPa with *T*_v_ = *T*_sat_ = 22.0 °C, *P*_v_ = 4.92 kPa with *T*_v_ = *T*_sat_ = 32.6 °C, and *P*_v_ = 10.41 kPa with *T*_v_ = *T*_sat_ = 46.6 °C, respectively. The light blue pentagrams are for *T*_v_ = 42.8 °C, *P*_v_ = 5.33 kPa with *T*_sat_ = 33.7 °C and the yellow hexagrams are for *T*_v_ = 45.5 °C, *P*_v_ = 6.11 kPa with *T*_sat_ = 36.5 °C. Note that the light blue pentagrams are also for a sample with a different porosity (≈0.23) and pore diameter (≈106 nm). The green dashed line, black dashed line, and blue dashed line are from the DSMC simulation with *σ* = 0.1, 0.31, and 0.5, respectively. The black solid line and black dotted line are from the HK equation and Schrage equation, respectively, both with *σ* *=* 0.31. The horizontal error bars originate from the uncertainties in temperature and pressure measurement (see Supplementary Notes 5 and 6). The error bars in *q̇̅*″ are smaller than the symbol size

Figure 5 shows the collapse of our results in both experiments and DSMC modeling with water when plotting *q̇̅″* as a function of Δ*P*/*P*_0_ for different working conditions. The red triangles, dark blue squares, light blue pentagram, yellow hexagrams, and orange circles represent the experimental data in a vapour ambient from Fig. 4, where we approximated *P*_K_ as *P*_v_ (which is more experimentally accessible) for low Mach numbers. More specifically, using continuum gas dynamics^13,14^, the pressure variation in the gas expansion region scales quadratically with *M*_K_ (see Supplementary Note 1). Meanwhile, based on previous literature^38^ as well as our DSMC modeling, the pressure drop across the Knudsen layer scales linearly with *M*_K_, which justifies our approximation. For the largest *M*_K_ (≈0.17) considered in this work, |*P*_v_ − *P*_K_|/ |*P*_0_ − *P*_K_| ≈ 0.014 (Supplementary Note 1). The dashed line represents the model fit with the DSMC calculation using the Levenberg-Marquardt nonlinear least squares algorithm^39^, which gives *σ* = 0.31 ± 0.03. The uncertainties mainly come from the error in the experimental data, where *σ* = 0.34 and *σ* = 0.28 correspond to the model fit with the minimum and maximum Δ*P*/*P*_0_ for each data point, respectively. We also plot the simulation results for *σ* = 0.1 and *σ* = 0.5 in Fig. 5 (green dashed line and blue dashed line) to show the sensitivity of the model to *σ*. This result of *σ* is based on our accurate interface sensing and consistent kinetic model, which is on the same order of magnitude as previous molecular dynamics studies^40,41^ and recent experimental results^15,20,42–45^ (see Supplementary Note 7 for more discussions). It is worthwhile mentioning that the light blue pentagrams are for a sample with a different porosity (≈0.23) and pore diameter (≈106 nm), which follows the same unifying curve. This indicates that the interfacial transport in each pore is not significantly affected by the porosity and the pore diameter. It also excludes the possibility of a thin liquid layer covering the membrane surface during operation, which is consistent with our previous studies^20,33^ and further justifies normalizing the evaporation heat transfer rate to the pore area (Eq. (1)).

## Discussion

Note that it is also possible to interpret the experimental data with the Hertz-Knudsen (HK) equation^46^ and the Schrage equation^47^ which are the two most commonly used evaporation models in literature. For small interfacial temperature difference, the HK equation is

7 $$\left[ {\dot q{\prime\prime}} \right]_{{\mathrm{HK}}} \,\, = \,\, \sigma \frac{{\Delta h_{{\mathrm{lv}}}}}{{\sqrt {2\pi RT_0} }}\Delta P$$

and the Schrage equation is

8 $$\left[ {\dot q{\prime\prime}} \right]_{\mathrm{S}} \,\, = \,\, \frac{{2\sigma }}{{2 - \sigma }} \cdot \frac{{\Delta h_{{\mathrm{lv}}}}}{{\sqrt {2\pi RT_0} }}\Delta P.$$

One can extract the same dimensionless flux and driving potential from Eqs. (7) and (8). We plot the model prediction from the HK equation and the Schrage equation with *σ* = 0.31 in Fig. 5 (black solid line and black dotted line). Although previous studies^48–50^ have pointed out that both these two evaporation models assume quasi-equilibrium conditions near the interface and do not conserve momentum and energy (the HK equation also violates mass balance), the results from Eqs. (7) and (8) do not differ much from the full BTE solution. This result, partly, is because we only considered low Mach number evaporation which is not far away from equilibrium conditions. Moreover, *σ* is relatively small in this work. When *σ* is close to unity, Eq. (7) and (8) provide two significantly different predictions ([*q̇″*]_S_ = 2 [*q̇″*]_HK_ for *σ* *=* 1). For a relatively small *σ*, [*q̇″*]_HK_ does not differ much from [*q̇″*]_S_ ([*q̇″*]_S_ = 1.18 [*q̇″*]_HK_ when *σ* *=* 0.31). Typically, the HK equation underestimates the interfacial heat flux whereas the Schrage equation leads to overestimation^50^. These results suggest that for low Mach numbers and small evaporation and condensation coefficients, neither the HK equation nor the Schrage equation generates much error when using Δ*P* as the driving potential.

In conclusion, we showed that evaporation kinetics can be described as the relationship between two dimensionless quantities: *q̇̅″* and Δ*P*/*P*_0_ by analyzing the BTE in the Knudsen layer. We experimentally demonstrated this fundamental relationship with evaporation of water into its own vapour. Experimental data at different working conditions are unified under this nondimensionalization scheme, which suggests that kinetic theory provides a proper modeling framework for evaporation problems. We used an ultrathin nanoporous membrane as the experimental platform to overcome the challenge of isolating the interfacial thermal resistance. We modeled the non-equilibrium gas kinetics with DSMC, where the rotational energy exchange was taken into account. This allowed for a more consistent interpretation of evaporation and condensation coefficients. We also showed that the commonly used HK equation and Schrage equation provide good approximations of the interfacial heat flux for low Mach numbers and small evaporation and condensation coefficients. We note that the current work is limited to low Mach numbers and only water was tested as the working fluid. Further investigation is needed to validate the theory for higher Mach number cases and other working fluids. Nevertheless, we demonstrated >300 W/cm^2^ evaporative heat fluxes (or >1.2 kg/m^2^s mass fluxes) with relatively low superheats (*T*_0_ − *T*_sat_ *<* 10 K). Our work offers design guidelines to improve evaporation performance of membrane-based cooling, steam generation, and desalination devices. More specifically, we showed that the ultrathin nanoporous configuration simultaneously minimizes thermal resistance and viscous loss while generating high capillary pressure, which enables rapid interfacial transport. Also, for evaporation systems operating in a pure vapour ambient, the unified relationship presented in this study can help determine the interfacial thermal resistance. Given a superheat constraint, our study suggests that *ρ*_0_*u*_s_Δ*h*_lv_ is a figure of merit that should be maximized when choosing working conditions and fluids.

## Methods

### Fabrication process

The ultrathin nanoporous membrane was microfabricated starting from a double side polished silicon wafer with both sides coated with silicon nitride (≈300 nm thick) using low pressure chemical vapour deposition. A nanopore array was patterned in the front silicon nitride layer using interference lithography and reactive ion etching (RIE) with tetrafluoromethane gas. The silicon nitride layer was not etched through, which protected the front side when the sample was etched from the back side using potassium hydroxide solutions. After that, two gold contact pads were deposited onto the sample with e-beam evaporation and shadow masking. Using another shadow mask, we etched through the pores from the front side with RIE and deposited a gold layer to serve as the resistive temperature detector (RTD) as well as the heater using e-beam evaporation. E-beam evaporation is a directional deposition process, so the gold deposition on the pore wall is minimal and does not block the pores. More details can be found in Supplementary Note 2.

### Experimental procedure

Prior to the experiments, the liquid reservoir tank was filled with deionized water (Water for HPLC, Sigma-Aldrich) and then heated to >100 °C for thermal degassing. The liquid reservoir was subsequently sealed from the ambient. Meanwhile, we calibrated the RTD to an industrial temperature sensor (P-L-A-1/4-6-1/4-T-6, Omega) in a convection oven (Supplementary Note 6). During the experiments, the environmental chamber was first pumped down to <0.5 Pa (confirmed by 925 Micro Pirani™ vacuum transducer, MKS) and then backfilled with pure water vapour from the reservoir. The vapour pressure in the chamber was regulated by the boiling canister temperature and measured by a capacitance pressure transducer (740C Baratron^®^ Manometer, MKS). The ambient vapour temperature was, on the other hand, set by the heating power put into the chamber wall and measured by five type K thermocouples placed at different locations in the chamber. We waited >2 h to ensure that the vapour ambient reached a steady state and the vapour temperature was uniform (within 1 °C based on the thermocouple readings). Using a peristaltic pump (UX-77921-77, Masterflex), we supplied liquid to the sample with the inlet flow rate maintained at 1 mL/min. We applied a four-point method to measure the total Joule heating power and obtain the interface temperature from the RTD. The measurements in this study were conducted in such a way that a set temperature was maintained. After setting the heating power to a higher value, the membrane temperature would increase, resulting in more intense evaporation at the interface. This served as a feedback loop as the cooling rate also increased. When the cooling rate matched the heating power, the system reached a steady state. We recorded the temperature and the heating power after maintaining the steady state for one minute. More details can be found in Supplementary Note 5.

### DSMC modeling

**Fig. 6:**
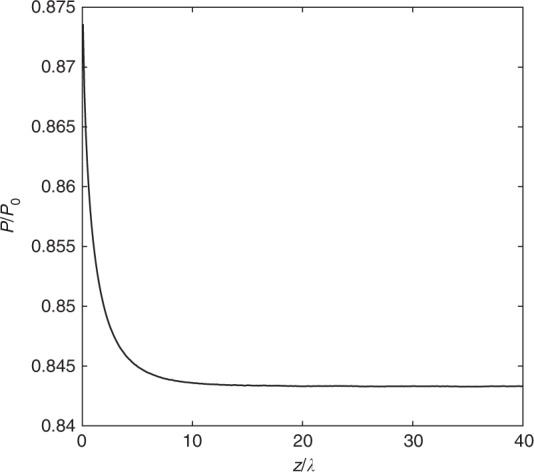
Vapour pressure *P* normalized to *P*_0_ as a function of the distance to the interface *z* normalized to the vapour mean free path for *u*_K_/*u*_s_ = 0.1 from DSMC simulation of the evaporation Knudsen layer

The DSMC method was used to solve the BTE in the Knudsen layer formed by the evaporation from a liquid surface. The collisions between water vapour molecules were handled using the variable soft sphere (VSS) collision model^35^ with a viscosity index *ω* = 1.047, scattering parameter *α* = 1.376, reference temperature *T*_ref_ = 350 K, and reference molecular diameter *d*_ref_ = 5.507 Å. These model parameters were determined from the temperature dependence of the viscosity, assuming that the Schmidt number coincides with the value for hard sphere gas molecules (Sc = 5/6). The equilibrium mean free path for VSS molecules can be calculated as^34^

9 $$\lambda = \frac{{\left( {T/T_{{\mathrm{ref}}}} \right)^{\omega - 0.5}}}{{\sqrt 2 \pi nd_{{\mathrm{ref}}}^2}}$$

where *T* and *n* are the vapour temperature and number density, respectively. The Borgnakke-Larsen method^36^ was implemented to reproduce the energy transfer between the translational and rotational degrees of freedom in molecular collisions. Based on the rotational collision numbers determined from the experiments^35,38^, the fraction of inelastic collisions for water vapour is between 0.2 and 0.4 for the temperature range that we considered. In this study, we set it to be 0.3 as it has been observed that the temperature/pressure ratio between the far field and the interface was not significantly affected by the choice of this value^36^. The typical size of the simulation cell was 10 nm, and each simulation particle represented to 5 × 10^13^ actual water molecules. The number of cells in the vertical direction *N*_z_ was chosen to ensure that the computational domain was large enough compared to the thickness of the Knudsen layer (*N*_z_ = 267–613, depending on the flow velocity). The collision steps in the DSMC calculations were processed following the no time counter algorithm^51^. The time step size of the simulation was 5 ps, and the sampling in each cell was performed after the flow reached the steady state. This method has shown good agreement with previous analysis of BTE of evaporation for monoatomic molecules^52^. The evaporation coefficient was defined as the ratio between the flux of molecules spontaneously escaping from the liquid and that drawn from a half-Maxwellian parameterized by *T*_0_ and *P*_0_, and the condensation coefficient was set as the probability of an incident vapour molecule condensing into the liquid phase instead of being reflected back. The reflection was assumed to be diffusive, as the interface can be considered to be rough on the molecular scale. We modeled evaporation of water for both *T*_sat_ = 40 and 80 °C and observed the collapse of simulation data when plotting *q̇̅*″ against Δ*P*/*P*_0_. Since the vapour is in nonequilibrium in the Knudsen layer, we evaluated the vapour pressure as^34^

10 $$P = \frac{1}{3}\mathop {\sum}\limits_{i = x,y,z} {{\int} {\left( {u_i - v_i} \right)^2\xi d{\mathbf{u}}} }$$

where *u*_*i*_ and *v*_*i*_ are components of the molecular velocity and the local flow velocity, respectively, and *ξ* is the mass-based distribution function. As an example, we plot the vapour pressure *P* normalized to *P*_0_ as a function of the distance to the interface *z* normalized to the vapour mean free path *λ* (evaluated at *T*_0_ and *P*_0_ based on Eq. (9)) for *u*_K_/*u*_s_ = 0.1 in Fig. 6. Generally, we calculate *P*_K_ only after *z*/*λ* > 20, and the calculation is insensitive to the exact value of the Knudsen layer thickness.

## Supplementary information

Supplementary Information

## Data Availability

All relevant data will be made available upon reasonable request from the authors.

## Code availability

The codes used this work are available upon reasonable request from the authors.
