# Supplementary material for: A unified relationship for evaporation kinetics at low Mach numbers
Source: Nat Commun. 2019 May 30;10:2368. doi: 10.1038/s41467-019-10209-w (PMC6542818; doi:10.1038/s41467-019-10209-w)
Supplement: Supplementary file 1 — Supplementary Information [file 41467_2019_10209_MOESM1_ESM.pdf]

Supplementary Information

A Unified Relationship for Evaporation Kinetics at Low Mach Numbers

Zhengmao Lu[a], Ikuya Kinefuchi[b], Kyle L. Wilke[a], Geoffrey Vaartstra[a], and Evelyn N. Wang[a*]

[a] Department of Mechanical Engineering, Massachusetts Institute of Technology, Cambridge, MA 02139, USA

[b] Department of Mechanical Engineering, University of Tokyo, Bunkyo, Tokyo 113-8656, Japan

[*] Corresponding author: enwang@mit.edu

Contents

Supplementary Figures 2

Supplementary Note 1: Vapour Transport Analysis 5

Supplementary Note 2: Fabrication Process 9

Supplementary Note 3: Device Design Rationale 10

Supplementary Note 4: Heat Loss Characterization 12

Supplementary Note 5: Experimental Procedure 13

Supplementary Note 6: RTD Calibration 15

Supplementary Note 7: Discussion on Evaporation and Condensation Coefficients 16

Supplementary Figures

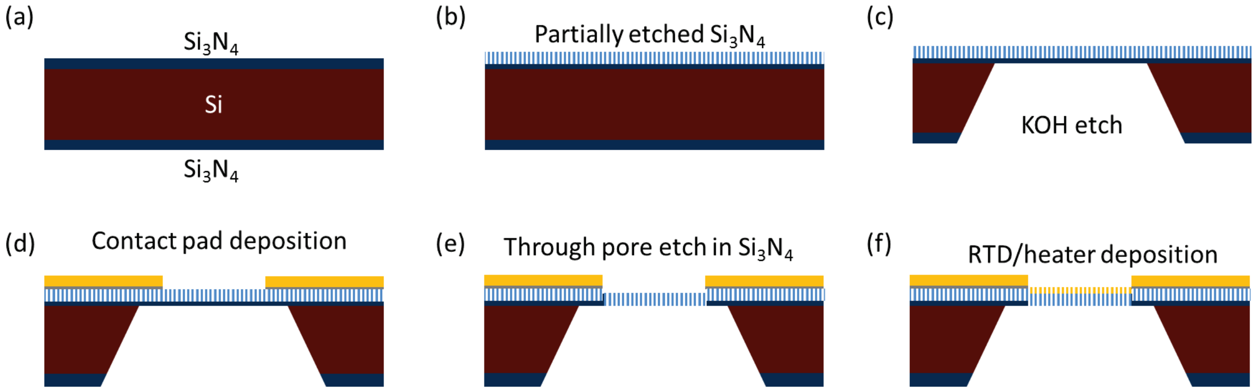

Supplementary Figure 1 Schematics of the fabrication process. (a) Low pressure chemical vapour deposition of silicon nitride on both sides of a double side polished silicon wafer. (b) Interference lithography on the front side to define nanoporous patterns which were partially etched into the silicon nitride layer via RIE. (c) Back etching using potassium hydroxide solutions with the front side protected due to the partial etch of the silicon nitride layer. (d) Contact pad deposition, (e) through pore etching and (f) RTD/heater deposition with shadow masking.

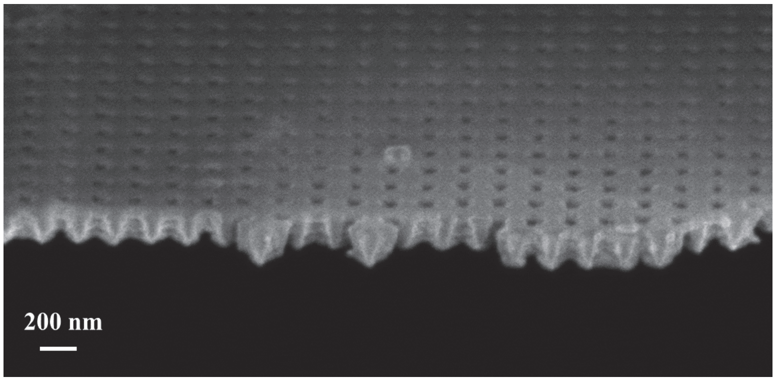

Supplementary Figure 2 SEM image of cross-section of the ultrathin nanoporous membrane at 15° tilting angle

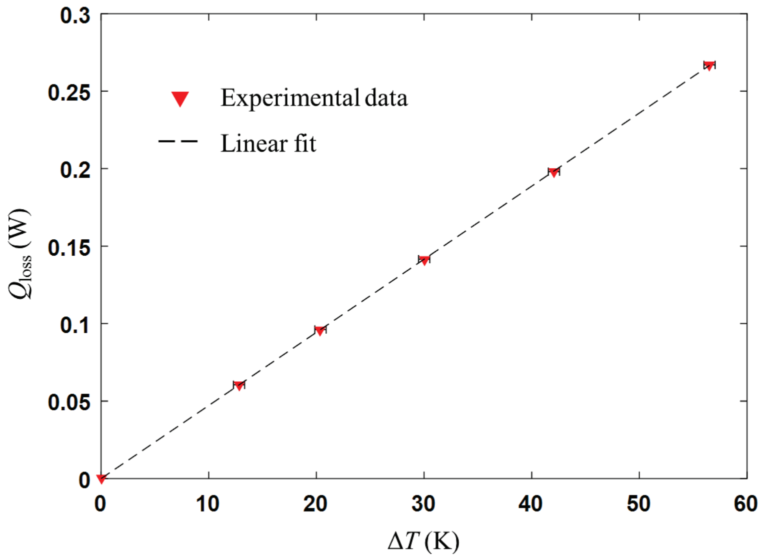

Supplementary Figure 3 Heat loss characterization using a control sample with an impermeable membrane. Q_{loss} as a function of ΔT and the experimental data (red triangles) were fitted to a linear model (black dashed line) $Q_{loss} = C\Delta T$ where $C = 4.7\pm0.1$ mW/K.

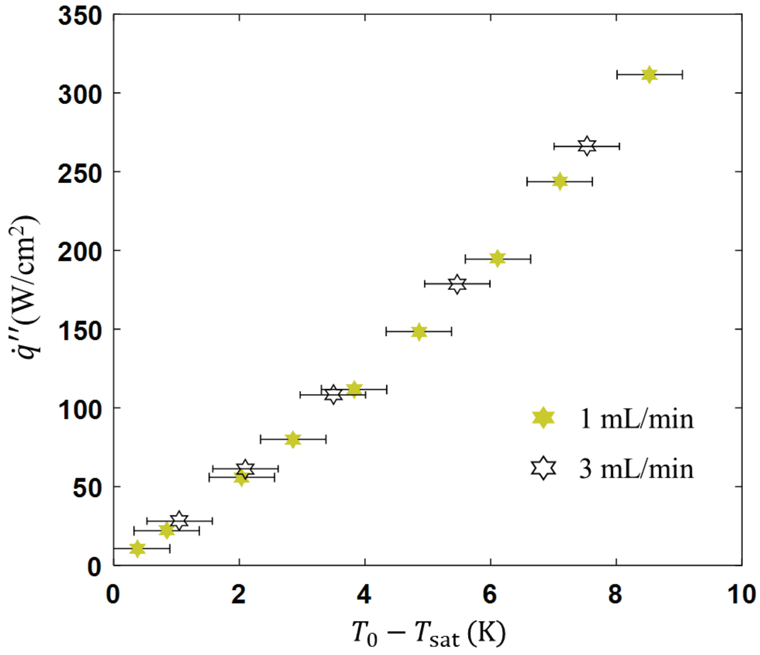

Supplementary Figure 4 Experimental results of $\dot{q}''$ as a function of $T_0 - T_{sat}$ for different flow rates.

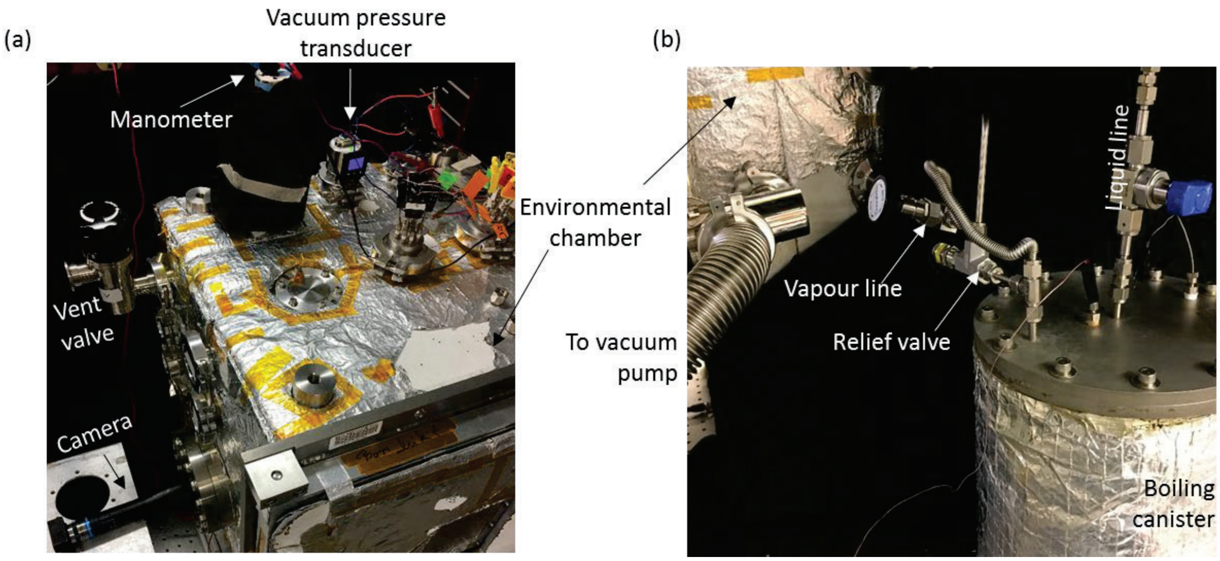

Supplementary Figure 5 (a) Environmental chamber that provides the pure vapour ambient (b) Boiling canister used as the liquid reservoir tank.

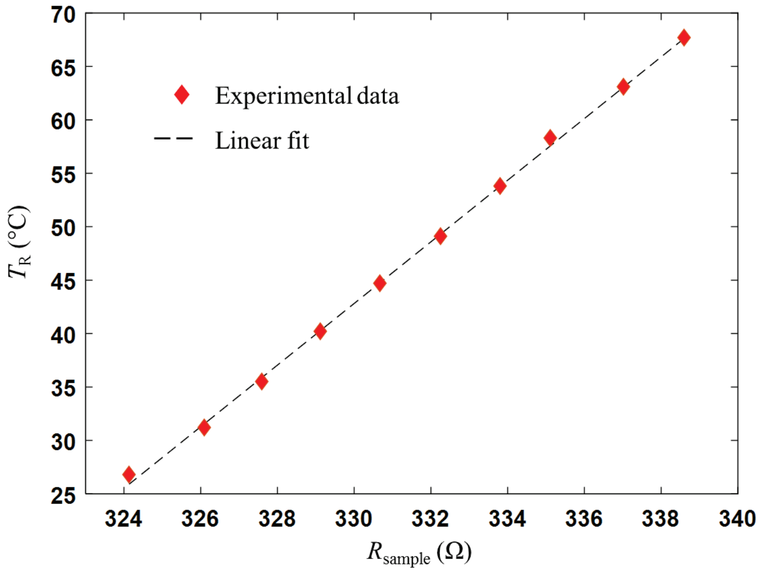

Supplementary Figure 6 Sample RTD calibration plot of T_R vs R_{sample}: the experimental data (red diamonds) were fitted to a linear model (black dashed line).

Supplementary Note 1: Vapour Transport Analysis

The gas kinetics in the Knudsen layer is governed by the Boltzmann Transport Equation (BTE).

$$\frac{\partial \xi}{\partial t} = -\mathbf{u} \cdot \nabla_{\mathbf{x}} \xi + \left[\frac{\partial \xi}{\partial t} \right]_{\text{collison}} \tag{1}$$

where ξ is the mass-based distribution function, t is time, and $\mathbf{u}$ is the molecular velocity. The first term on the right hand side represents the streaming of molecules and the second term is the change of distribution function due to collisions between molecules. These two terms both contribute to the time evolution of ξ (the left hand side). Choosing the reference temperature to be the interface temperature T_0, the reference sonic speed is then given by

$$u_{\text{s}} = \sqrt{\gamma R T_0} \tag{2}$$

where γ is the ratio of specific heats and R is the specific gas constant. The reference pressure is set as P_0, the saturation pressure at T_0 and the reference density is set as ρ_0, the saturation vapour density at T_0.

We can nondimensionalize the distribution function as

$$\tilde{\xi}\left(\tilde{\mathbf{u}}, \tilde{\mathbf{x}}, \tilde{t}\right) = \frac{u_s^{\ 3}}{\rho_0} \xi\left(\mathbf{u}, \mathbf{x}, t\right) \tag{3}$$

where $\tilde{\mathbf{u}} = \mathbf{u}/u_{\text{s}}$, $\tilde{\mathbf{x}} = \mathbf{x}/l_0$ with l_0 being the characteristic length, and $\tilde{t} = t/\tau$ with τ being the characteristic relaxation time for molecular collisions. Accordingly, in the dimensionless form, the BTE can be rewritten as:

$$\frac{\partial \tilde{\xi}}{\partial \tilde{t}} = -\frac{u_{\text{s}} \tau}{l_0} \tilde{\mathbf{u}} \cdot \nabla_{\tilde{\mathbf{x}}} \tilde{\xi} + \left[\frac{\partial \tilde{\xi}}{\partial \tilde{t}} \right]_{\text{collison}} \tag{4}$$

where $u_{\text{s}}\tau/l_0$ is proportional to the Knudsen number.

The vapour distribution function at the interface ξ_0 can be written as[1, 2, 3]:

$$\xi_0 \,|_{u_z>0} = \left[\sigma_e \rho_0 + (1-\sigma_c)\rho_\mathrm{r}\right] \frac{\exp\left[-\mathbf{u}^2/2RT_0\right]}{(2\pi RT_0)^{3/2}} \tag{5}$$

where u_z is its z-component of the molecular velocity and ρ_r is the reference density of vapour molecules approaching the interface[1]:

$$\rho_\mathrm{r} = \frac{\int_{u_z<0} |u_z| \xi_0 d\mathbf{u}}{\sqrt{RT_0/2\pi}} \tag{6}$$

After nondimensionalization,

$$\tilde{\xi}_0 \,|_{\tilde{u}_z>0} = \left[\sigma_e + (1-\sigma_c)\tilde{\rho}_\mathrm{r}\right] \left(\frac{\gamma}{2\pi}\right)^{3/2} \exp\left(-\frac{\gamma \tilde{\mathbf{u}}^2}{2}\right) \tag{7}$$

where

$$\tilde{\rho}_\mathrm{r} = \frac{\rho_\mathrm{r}}{\rho_0} = \sqrt{2\pi\gamma} \int_{\tilde{u}_z<0} |\tilde{u}_z| \tilde{\xi}_0 d\tilde{\mathbf{u}} \tag{8}$$

The vapour flow coming out of the Knudsen layer follows a drifted Maxwell-Boltzmann distribution, where we assume local thermodynamic equilibrium:

$$\xi_\mathrm{K} = \rho_\mathrm{K} \frac{\exp\left[-(\mathbf{u} - u_\mathrm{K}\hat{\mathbf{z}})^2/2RT_\mathrm{K}\right]}{(2\pi RT_\mathrm{K})^{3/2}} \tag{9}$$

where T_K is the temperature at the boundary of the Knudsen layer. After nondimensionalization, we can rewrite Supplementary Eq. (9) as

$$\tilde{\xi}_\mathrm{K} = \frac{\rho_\mathrm{K}}{\rho_0} \left(\frac{\gamma T_0}{2\pi T_\mathrm{K}}\right)^{3/2} \exp\left[-\left(\tilde{\mathbf{u}} - \frac{u_\mathrm{K}}{u_\mathrm{s}}\hat{\mathbf{z}}\right)^2 \left(\frac{\gamma T_0}{2T_\mathrm{K}}\right)\right] \tag{10}$$

Assuming ideal gas behavior, we have

$$\frac{\rho_{\mathrm{K}}}{\rho_0} = \frac{P_{\mathrm{K}}}{P_0} \bigg/ \frac{T_{\mathrm{K}}}{T_0} \tag{11}$$

and
$$\frac{u_{\mathrm{K}}}{u_s} = M_{\mathrm{K}} \sqrt{\frac{T_{\mathrm{K}}}{T_0}}. \tag{12}$$

where M_{K} is the Mach number at the Knudsen boundary and $M_{\mathrm{K}} = u_{\mathrm{K}}/(\gamma R T_{\mathrm{K}})^{1/2}$.

From the Eqs. (7), (8), and (10)-(12), we observe that the boundary conditions of Supplementary Eq. (4) are uniquely determined by P_{K}/P_0, T_{K}/T_0, and M_{K}. Previous studies[1, 2, 3, 4] also recognized that these are the three key parameters that characterize the Knudsen layer problem. More specifically, it was demonstrated that for evaporation problems, one of the three parameters uniquely determines the other two. In other words, each of the three parameters can be considered as a function of $\Delta P/P_0$ where $\Delta P = P_0 - P_{\mathrm{K}}$. Equations (3), (4), and (5) in the main text shows that $\bar{q}''$ is a function of P_{K}/P_0, T_{K}/T_0, and M_{K} and thus can also be determined from $\Delta P/P_0$. Previous studies showed that ΔP scales linearly with M_{K}.[2, 4, 5] Meanwhile, for the gas expansion region, using the isentropic approximation, we can write

$$\frac{P_{\mathrm{v}} - P_{\mathrm{K}}}{P_{\mathrm{K}}} = \left(1 + \frac{\gamma - 1}{2} M_{\mathrm{K}}^{2}\right)^{\frac{\gamma}{\gamma-1}} - 1 \tag{13}$$

P_{v} is the pressure measured >0.1 m away from the interface in the chamber whereas the expected vapour mean free path varied between 0.29 μm and 1.1 μm for different working conditions following Supplementary Eq. (7) in the main text. As the chamber characteristic size (~0.1 m) is much larger than the evaporator characteristic size (~0.1 mm), the vapour flow expands significantly and decelerates to almost 0 speed once it is far away from the evaporating surface. Therefore, P_{v} can be considered as the stagnation vapour pressure. For small Mach numbers, the right hand side of Supplementary Eq. (13) can be approximated as $\gamma M_{\mathrm{K}}^2/2$. Therefore, the pressure variation in the gas expansion region scales with M_{K}^2. On the other hand, based on the DSMC simulation, the pressure drop across the Knudsen layer scales linearly with M_{K}. This justifies the $P_{\mathrm{K}} = P_{\mathrm{v}}$ approximation for small Mach numbers. For the largest M_{K} (≈0.17)

considered in this work, we have $|P_\mathrm{v}-P_\mathrm{K}|/|P_0-P_\mathrm{K}| \approx 0.014$, where $|P_\mathrm{v}-P_\mathrm{K}|$ is calculated from Supplementary Eq. (13) and $|P_0-P_\mathrm{K}|$ is obtained from the DSMC simulation.

Supplementary Note 2: Fabrication Process

The ultrathin nanoporous membrane was microfabricated starting from a double side polished silicon wafer with both sides coated with silicon nitride (≈300 nm thick) using low pressure chemical vapour deposition (Supplementary Figure 1a). A nanopore array was patterned in the front silicon nitride layer using interference lithography and reactive ion etching (RIE) with tetrafluoromethane gas (Supplementary Figure 1b). The silicon nitride layer was not etched through, which protected the front side when the sample was etched from the back side using potassium hydroxide solutions (Supplementary Figure 1c). After that, two gold contact pads were deposited onto the sample with e-beam evaporation and shadow masking (Supplementary Figure 1d). Using another shadow mask, we etched through the pores from the front side with RIE and deposited a gold layer to serve as the resistive temperature detector (RTD) as well as the heater (Supplementary Figure 1e-f) using e-beam evaporation. E-beam evaporation is a directional deposition process, so the gold deposition on the pore wall is minimal and does not block the pores. Supplementary Figure 2 shows the cross-section image of the ultrathin nanoporous membrane. Also, since the contact angle of water on gold is below 90° [6], liquid can still wick into the pore from the bottom and wet along the pore wall, and the meniscus is pinned at the top corner of the pore given some slight gold deposition on the pore wall.

Supplementary Note 3: Device Design Rationale

We identified several experimental challenges for characterizing the interfacial transport which include: (1) measuring the interface temperature accurately and non-invasively; (2) decoupling the interfacial thermal resistance from the thermofluidic resistance in the liquid phase and the diffusion resistance in the vapour phase; and (3) mitigating the risk of blockage of evaporating surface due non-evaporative contaminants.

The temperature variation along the interface δT_0 can be estimated as

$$\delta T_0 = \dot{q}'' \frac{L}{k} \tag{14}$$

where $\dot{q}''$ is the interfacial heat flux, k is the thermal conductivity of the working fluid, and L is the characteristic length for thermal conduction in the liquid (estimated as the pore radius in this case). For a reference condition where $\dot{q}'' = 100$ W/cm^2, $k = 0.6$ W/m-K (for water), and $L = 70$ nm, $\delta T_0 = 0.12$ K. This indicates that the pore diameter is small enough for the thermal resistance in the liquid to be negligible and the temperature of the gold layer is approximately the same as the interface, which allows us to measure the interface temperature accurately and non-invasively.

The pressure drop along the pore can be estimated using the Hagen–Poiseuille equation:

$$P_{\mathrm{vis}} = \frac{32\mu_{\mathrm{l}} t_{\mathrm{m}} \dot{q}''}{\pi d_{\mathrm{p}}^{2} \rho_{\mathrm{l}} \Delta h_{\mathrm{lv}}} \tag{15}$$

where μ_{l} is the liquid viscosity, t_{m} is the membrane thickness, ρ_{l} is the liquid density, and Δh_{lv} is the enthalpy difference between the two phases. Setting $\dot{q}'' = 100$ W/cm^2, $t_{\mathrm{m}} = 200$ nm, $d_{\mathrm{p}} = 140$ nm, and using properties of water at the room temperature, we have $P_{\mathrm{vis}} < 100$ Pa. This is much smaller than the characteristic capillary pressure $\gamma_{\mathrm{lv}}/d_{\mathrm{p}}$ where γ_{lv} is the surface tension. During operation, the interface is pinned at the top corner of the pore, creating a self-regulating system, i.e., the apparent contact angle on the pore wall (or the curvature of the interface) adjusts to the actual interfacial pressure difference. The very small pressure difference across the interface implies that the interface is almost flat during evaporation.

The ultrathin membrane also mitigates the clogging risk which nanoporous configurations are often prone to. The mass fraction of non-evaporative contaminants in the nanopore $c(z)$ is governed by the 1-D steady state convection-diffusion equation:

$$-D_{\mathrm{c}}\frac{dc}{dz}+v_{\mathrm{liq}}c=0 \tag{16}$$

where D_{c} is the diffusion coefficient of the contaminant in liquid water (~ 10^{-5} cm^2/s) and v_{liq} is the volume flux of the liquid flow in the nanopore ($v_{\mathrm{liq}} \approx 0.05$ cm/s for $\dot{q}'' = 100$ W/cm^2). Supplementary Eq. (16) yields that

$$c_{\mathrm{in}}=c_0\exp\left(\frac{v_{\mathrm{liq}}t_{\mathrm{m}}}{D_{\mathrm{c}}}\right) \tag{17}$$

where c_{in} and c_0 are the concentrations of contaminants at the interface and in the flushing flow underneath the membrane, respectively, and t_{m} is the membrane thickness. The Péclet number of the system can be defined as $\mathrm{Pe} = v_{\mathrm{liq}}\, t_{\mathrm{m}} / D_{\mathrm{c}}$. The contamination level at the interface increases sharply with larger Pe and the thinner the membrane, the smaller Pe is. For $\dot{q}'' = 100$ W/cm^2, with $t_{\mathrm{m}} \approx 200$ nm and $c_0 \sim 3\times10^{-6}$ (Water for HPLC, Sigma-Aldrich), we have $\mathrm{Pe} \sim 0.1$ and $c_{\mathrm{in}} \sim 3.3\times10^{-6}$ which ensures a clean (<0.001% contamination) liquid-vapour interface during evaporation.

Supplementary Note 4: Heat Loss Characterization

To characterize the heat loss of the system, we fabricated a control sample which had the same structure as the designed device except that the active part was impermeable, *i.e.*, had no pores. With liquid supplied at 1 mL/min (same as in the evaporation experiment), we measured the heating power Q_{loss} as a function of the temperature rise of the membrane of the control sample ΔT as the sample was cooled down by convection and conduction. Q_{loss} is plotted as a function of ΔT in Supplementary Figure 3, and the experimental data (red triangles) are fitted to a linear model (black dashed line) $Q_{\text{loss}} = C\Delta T$ where C = 4.7±0.1 mW/K. The heat loss conductance without no inlet liquid flow was measured to be 0.42 ±0.2 mW/K, indicating convection is the main heat loss mechanism in our study.

To further test the effect of the inlet flow rate, for the T_{v} = 45.5 °C, T_{sat} = 36.5 °C case, we also performed the experiment at both 1 mL/min and 3 mL/min inlet flow rates with data shown in Supplementary Figure 4, where the results does not vary much as the inlet flow rate is changed. The heat loss was small in both cases ($C \approx 6.4 \pm 0.2$ mW/K for 3 mL/min inlet flow rates).

Supplementary Note 5: Experimental Procedure

The environmental chamber that we used in this study was equipped with liquid feedthroughs, electrical feedthroughs, thermocouple feedthroughs, viewports, and pressure transducers (Supplementary Figure 5a). It was connected to a rotary vane vacuum pump and a boiling canister which was used as the liquid reservoir tank in the current work (Supplementary Figure 5b). Prior to the experiments, the liquid reservoir tank was filled with deionized water (Water for HPLC, Sigma-Aldrich) and then heated to >100 °C for thermal degassing. The liquid reservoir was subsequently sealed from the ambient. Meanwhile, we calibrated the RTD to an industrial temperature sensor (P-L-A-1/4-6-1/4-T-6, Omega) in a convection oven. During the experiments, the environmental chamber was first pumped down to <0.5 Pa (confirmed by 925 Micro Pirani™ vacuum transducer, MKS) and then backfilled with pure water vapour from the reservoir. The vapour pressure in the chamber was regulated by the boiling canister temperature and measured by a capacitance pressure transducer (740C Baratron® Manometer, MKS). The ambient vapour temperature was, on the other hand, set by the heating power put into the chamber wall and measured by five type K thermocouples placed at different locations in the chamber. We waited > 2 hours to ensure that the vapour ambient reached a steady state and the vapour temperature was uniform (within 1 °C based on the thermocouple readings). Using a peristaltic pump (UX-77921-77, Masterflex), we supplied liquid to the sample with the inlet flow rate maintained at 1 mL/min. We applied a four-point method to measure the total Joule heating power and obtain the interface temperature from the RTD. The measurements in this study were conducted in such a way that a set temperature was maintained. After setting the heating power to a higher value, the membrane temperature would increase, resulting in more intense evaporation at the interface. This served as a feedback loop as the cooling rate also increased. When the cooling rate matched the heating power, the system reached a steady state. We recorded the temperature and the heating power after maintaining the steady state for one minute. On the other hand, due to the ultralow thermal mass of the membrane, the thermal time constant τ was very small:

$$\tau = \frac{mc_{\mathrm{p}}}{UA} \tag{18}$$

where m is the mass of the membrane, c_{p} is its specific heat, A is the interface area, and U is the overall heat transfer coefficient. In our setup, $m < 10^{-8}$ kg, $c_{\mathrm{p}} < 2$ J/g-K, and $UA \gg C$, such that $\tau < 0.01$ s. The heating power in the experiment was limited by the onset of nucleation beneath the membrane. Due to the low hydraulic resistance across the thin membrane separating the two phases, the liquid and vapour were at similar pressures during operation. Although most heat was dissipated through evaporation, the membrane temperature was still elevated as we increased the heat fluxes, resulting in superheated liquid adjacent to the membrane. As we approached larger superheats with higher heating powers, cavitation became more likely to occur in the metastable liquid, which would cause the mechanical failure of the membrane. The experimental data that we reported in this study corresponded to the largest superheats we achieved under each working condition without observing the onset of bubble nucleation.

Supplementary Note 6: RTD Calibration

Prior to the experiment, the resistance of the gold layer was calibrated to an industrial temperature sensor (P-L-A-1/4-6-1/4-T-6, Omega) in a gravity convection oven (1370GM, VWR). We set the oven temperature with its own controller and waited for >2 hours for thermal equilibrium in the oven. The resistance of the gold layer (R_{sample}) and the reference temperature sensor reading (T_R) were both obtained by a data acquisition device (9226, National Instruments) with a four-wire method. A sample calibration curve is shown in Supplementary Figure 6. We fit a linear model (black dashed line) to the experimental data. The industrial temperature sensor has an error of δT_1 = 0.25 K for the temperature range considered in the present study, and the linear fit causes an additional error of δT_2 = 0.39 K. The overall temperature measurement error is then $\delta T = (\delta T_0^2 + \delta T_1^2 + \delta T_2^2)^{1/2} \approx 0.52$ K.

Supplementary Note 7: Discussion on Evaporation and Condensation Coefficients

The dashed line in Figure 5 in the main text represents the least-square model fit with equal evaporation and condensation coefficients where $\sigma_e = \sigma_c = \sigma = 0.31 \pm 0.03$. Molecular dynamics simulations of water generally yielded the same order of magnitude. Although various values have been reported[6, 7, 8], which may be due to different intermolecular potential models used, the simulation results generally suggest no significant variation of σ_e and σ_c for the temperature range that we considered. Previous experimental studies, on the other hand, reported σ_e and σ_c across three different orders of magnitude (0.002-1)[9]. Restricting the comparison to the studies with dynamically renewing interfaces[10, 11, 12, 13, 14], the results become more similar to the current work (0.1-1), indicating contamination could be a severe challenge in many previous studies. There are several transient evaporation studies with liquid water exposed to vacuum, which simplifies the vapour transport into the free molecular flow. Hickman[8] studied evaporation into vacuum from falling water, with only bulk liquid temperature measurements using thermocouples and obtained σ_e = 0.254-0.532. Winkler et al.[12] estimated σ_e and σ_c as 0.4-1 based on the droplet growth kinetics in an expansion cloud chamber without interface temperature measurement. More recently, Smith et al. used Raman thermometry to probe the surface temperature of evaporating water droplet in vacuum and reported $\sigma_e = 0.62 \pm 0.09$[15]. However, their thermal model did not account for the size change of the droplet and the large Biot number of the droplet. For vapour ambient studies, Narusawa and Springer[13] measured the evaporation rate of water in a cylindrical container in a vacuum chamber, determined the interface temperature with thermal radiation, and reported $\sigma_e = 0.19$. Nevertheless, the Schrage model that they used is known to overpredict the interfacial flux due to violation of momentum and energy conservation[16], which means their σ_e should be higher than what they reported. Kobayashi et al.[15] reported σ_c = 0.71-0.84 with shock tube experiments and numerical simulations of the Gaussian-BGK Boltzmann equation. Since their model did not account for the energy exchange due to the rotational modes of water molecules and underpredicted the interfacial heat flux, their σ_c should, in fact, be smaller than what they reported. In general, even though the present work obtained similar σ_e and σ_c compared to many previous studies, it represents quite different interfacial heat

transfer as the Knudsen layer non-equilibrium, and internal degrees of freedom of water molecules were generally not properly taken into account when interpreting the experimental data in prior works.

Supplementary References

1. Frezzotti A. Boundary conditions at the vapor-liquid interface. *Phys Fluids* **23**, 030609 (2011).

2. Sone Y, Takata S, Golse F. Notes on the boundary conditions for fluid-dynamic equations on the interface of a gas and its condensed phase. *Phys Fluids* **13**, 324-334 (2001).

3. Meland R, Frezzotti A, Ytrehus T, Hafskjold B. Nonequilibrium molecular-dynamics simulation of net evaporation and net condensation, and evaluation of the gas-kinetic boundary condition at the interphase. *Phys Fluids* **16**, 223-243 (2004).

4. Meland R, Ytrehus T. Evaporation and condensation Knudsen layers for nonunity condensation coefficient. *Phys Fluids* **15**, 1348-1350 (2003).

5. Labuntsov D, Kryukov A. Analysis of intensive evaporation and condensation. *Int J Heat Mass Transfer* **22**, 989-1002 (1979).

6. Frezzotti A. A numerical investigation of the steady evaporation of a polyatomic gas. *European Journal of Mechanics-B/Fluids* **26**, 93-104 (2007).

7. Smith T. The hydrophilic nature of a clean gold surface. *J Colloid Interface Sci* **75**, 51-55 (1980).

8. Wilke KL, Barabadi B, Lu Z, Zhang T, Wang EN. Parametric study of thin film evaporation from nanoporous membranes. *Appl Phys Lett* **111**, 171603 (2017).

9. Yang T, Pan C. Molecular dynamics simulation of a thin water layer evaporation and evaporation coefficient. *Int J Heat Mass Transfer* **48**, 3516-3526 (2005).

10. Tsuruta T, Nagayama G. Molecular dynamics studies on the condensation coefficient of water. *J Phys Chem B* **108**, 1736-1743 (2004).

11. Ishiyama T, Yano T, Fujikawa S. Molecular dynamics study of kinetic boundary condition at an interface between a polyatomic vapor and its condensed phase. *Phys Fluids* **16**, 4713-4726 (2004).

12. Marek R, Straub J. Analysis of the evaporation coefficient and the condensation coefficient of water. *Int J Heat Mass Transfer* **44**, 39-53 (2001).

13. Hickman K. Maximum evaporation coefficient of water. *Industrial & Engineering Chemistry* **46**, 1442-1446 (1954).

14. Lee J, Laoui T, Karnik R. Nanofluidic transport governed by the liquid/vapour interface. *Nat Nanotechnol* **9**, 317 (2014).

15. Smith JD, Cappa CD, Drisdell WS, Cohen RC, Saykally RJ. Raman thermometry measurements of free evaporation from liquid water droplets. *J Am Chem Soc* **128**, 12892-12898 (2006).

16. Winkler PM, Vrtala A, Wagner PE, Kulmala M, Lehtinen KEJ, Vesala T. Mass and Thermal Accommodation during Gas-Liquid Condensation of Water. *Phys Rev Lett* **93**, 075701 (2004).
